# A Case Report on Primary Central Nervous System Lymphoma in Immunocompetent Individual

**DOI:** 10.7759/cureus.15990

**Published:** 2021-06-28

**Authors:** Shobha Mandal, Sunil Shah, Sumit Gami, Barun Ray, Joyson Poulose

**Affiliations:** 1 Internal Medicine, Guthrie Robert Packer Hospital, Sayre, USA; 2 Medicine, Ministry of Health, Malé, MDV; 3 Medicine, California Institute of Behavioral Neurosciences & Psychology, Fairfield, USA; 4 Medicine, Universal College of Medical Sciences, Bhairahawa, NPL; 5 Internal Medicine, Nidan Hospital, Kathmandu, NPL; 6 Internal Medicine, B.P. Koirala Institute of Health Sciences, Dharan, NPL; 7 Hematology and Oncology, Guthrie Robert Packer Hospital, Sayre, USA

**Keywords:** primary cns lymphoma, ataxia, diffuse large b cell lymphoma, pcnsl, immunosuppression

## Abstract

Primary central nervous system lymphoma (PCNSL) is a rare form of CNS tumor. Immunosuppression is the most important risk factor of PCNSL, but few immunocompetent individuals are also affected. The most common clinical feature of PCNSL includes dizziness and ataxia. Our patient was a 71-year-old immunocompetent female who presented with progressive ataxia, imbalance, and dizziness for one year. A homogenous enhancing lesion was found in magnetic resonance imaging (MRI) of the head. One month later, on a follow-up MRI, a significant increase in the tumor size with surrounding edema was seen. The patient underwent craniotomy and resection of the mass. Subsequently, a diagnosis of diffuse large B cell lymphoma was made on pathology examination. Guideline-directed treatment options were discussed. This case highlights that the prognosis of patients with PCNSL highly depends on the factors such as age and physical status. Early diagnosis by accurate interpretation of imaging and management is crucial for better health outcomes.

## Introduction

Primary central nervous system lymphoma (PCNSL) is one of the rare forms of brain tumor. PCNSL mostly includes B-cell lymphomas (approximately 90%) that account for 2% to 3% of all brain tumors. Less than 1% of all non-Hodgkin lymphomas are PCNSLs [1]. The only known risk factor for this disease is immune dysfunction. Patients with congenital immune dysfunction such as IgA deficiency, Wiskott-Aldrich syndrome, X-linked lymphoproliferative syndrome, and acquired immunosuppression such as human immunodeficiency virus (HIV)/acquired immunodeficiency syndrome (AIDS), post-bone marrow, and solid-organ transplantation show increased incidence of PCNSL. Among these, HIV/AIDS is the most important risk factor [2]. We report a rare case of PCNSL in a 71-year-old immunocompetent female to provide significant clinical insights over the importance of age, physical status, and the early accurate diagnosis of PCNSL for a better prognosis.

## Case presentation

**Figure 1:**
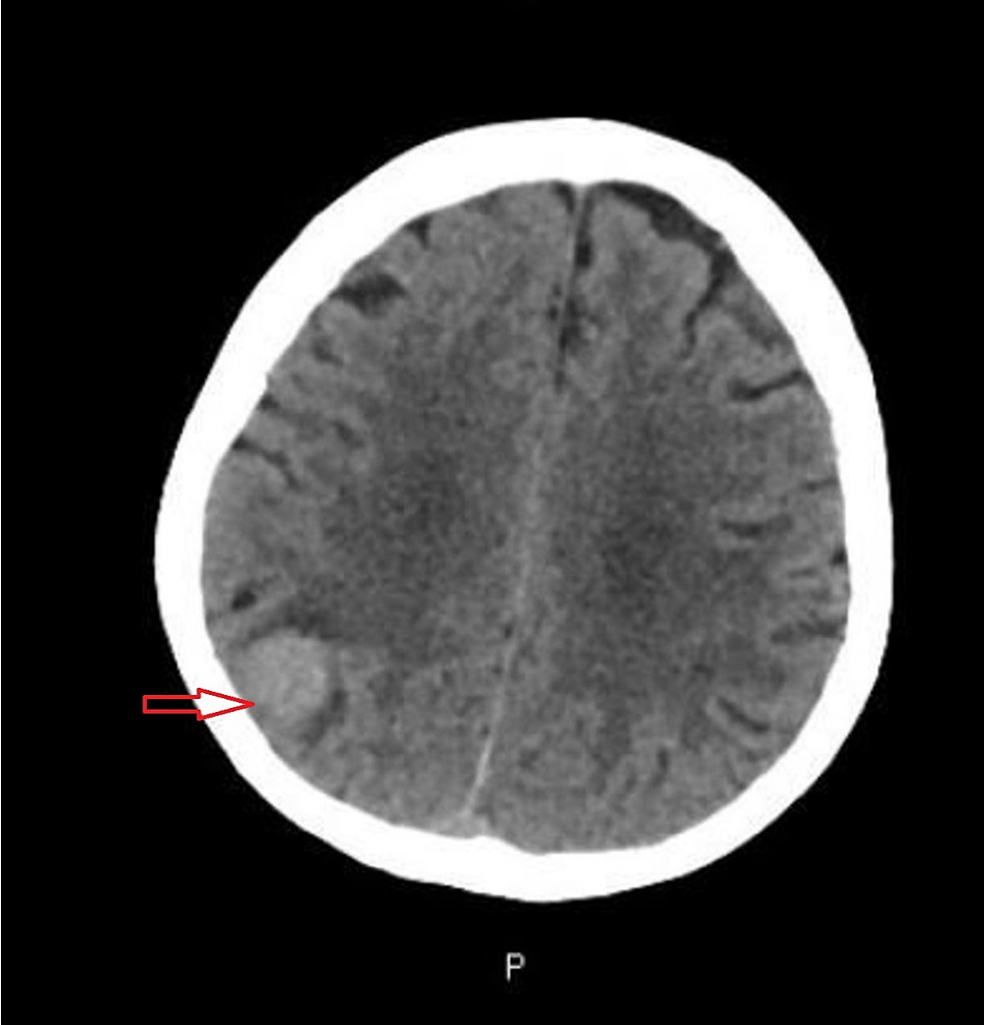
CT Head (Axial View) Showing Hyperdense Lesion in the Right Posterior Parietal Lobe

**Figure 2:**
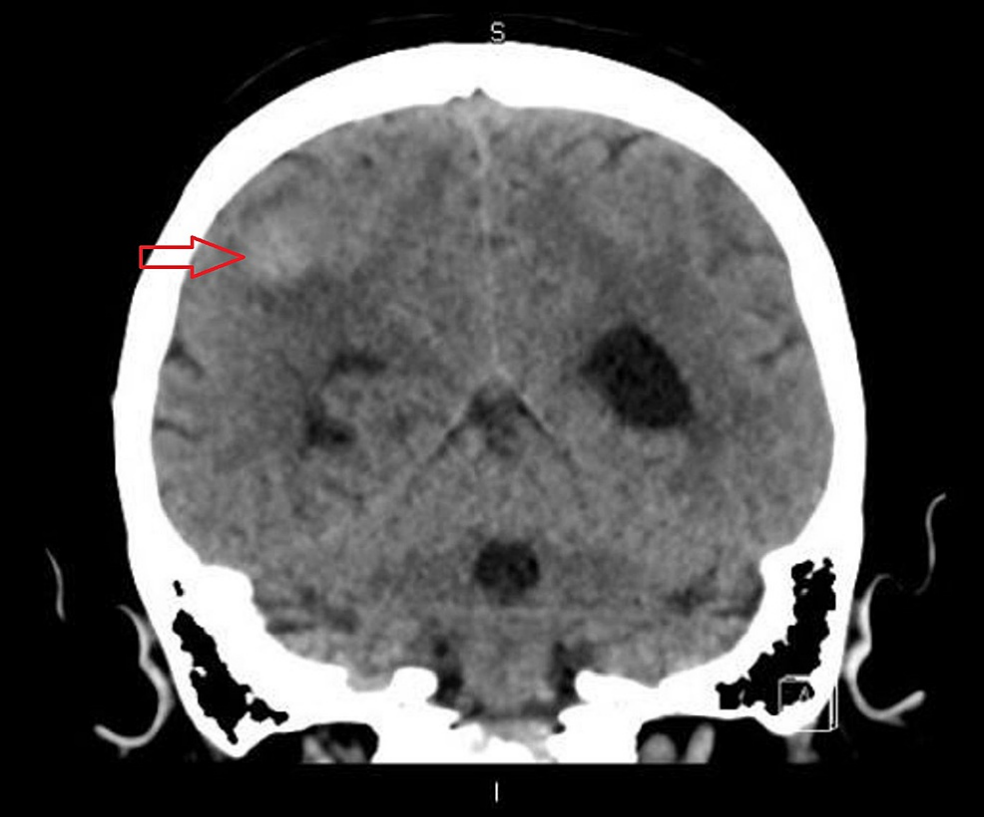
CT Head (Coronal View) Showing Hyperdense Lesion in the Right Posterior Parietal Lobe

A 71-year-old female presented to our hospital with gradually worsening balance, ataxia, and dizziness for one year. Six months later she started having a new-onset headache which was progressively getting worse, was not relieved with over-the-counter medications. Headache was associated with loss of peripheral vision without any focal weakness, dysphagia, bladder or bowel incontinence, irritability, and apathy. She came to the emergency department for evaluation. Vital signs including body temperature, blood pressure, and heart rate were within normal limits. On neurological examination muscle strength was 4 out of 5 in all four extremities, gait was impaired, and rest examination was normal. Computed tomography (CT) scan of the brain demonstrated a hyperdense focus within the right posterior parietal lobe (Figure 1 and Figure 2).

**Figure 3:**
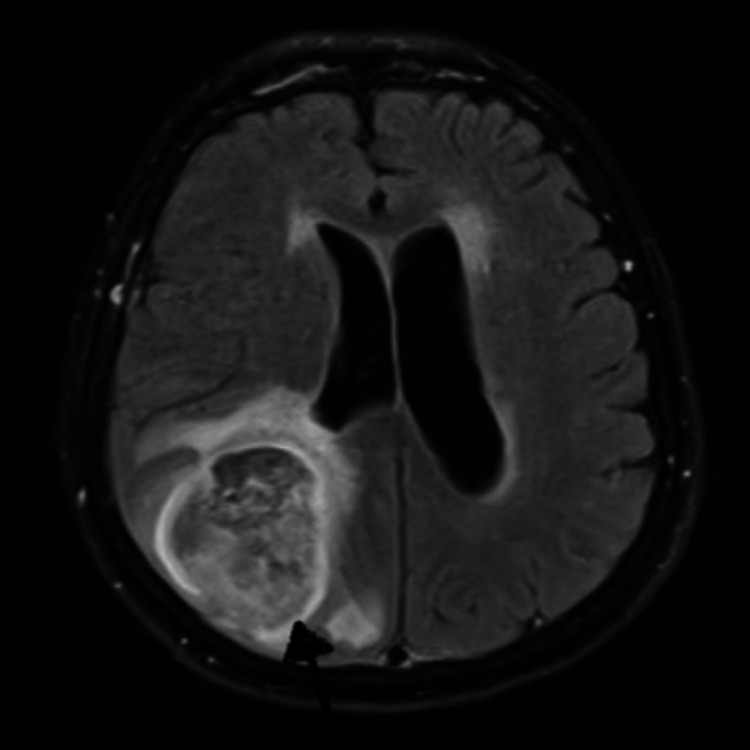
MRI (Axial View) Showing the Homogenous Lesion in the Right Parietal Lobe

This was followed up with a magnetic resonance imaging (MRI) of the brain that demonstrated a new homogeneously enhancing lesion abutting the grey-white matter of the right parietal lobe concerning brain metastases versus primary brain neoplasm. Therefore, extensive imaging was carried out to look for extracranial disease and distant metastases. A CT scan of the chest, abdomen, and pelvis was normal. On evaluation by the neurosurgery team, the patient was advised to undergo a brain mass biopsy to confirm the diagnosis. She refused the biopsy; hence she was presumed to have a benign tumor and was followed up with a repeat MRI in one month. A repeat MRI two months later showed significant interval enlargement of the lesion with significant edema surrounding the lesion along with interval development of hemorrhage (Figure 3).

**Figure 4:**
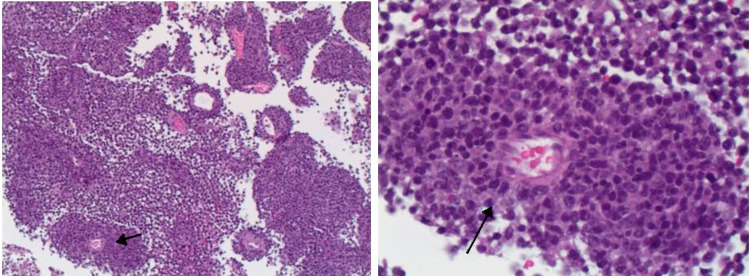
Immunohistochemistry Showing a Perivascular Collection of Large Atypical Lymphocytes, H&E, X100 and X400

As there was significant enlargement of the tumor over the course of two months, and she had a left hemianopsia with deficit to the right visual field. Hence patient was admitted and underwent right craniotomy with resection of the tumor. She had no immediate post-operative complications. Histopathology was confirmatory for diffuse large B-cell lymphoma, non-germinal center B-cell type (Figure 4). Fluorescence in situ hybridization (FISH)/cytogenetics was negative for CD10, MYC, and IGH-BCL2 fusion but positive for rearrangement of BCL2.

**Figure 5:**
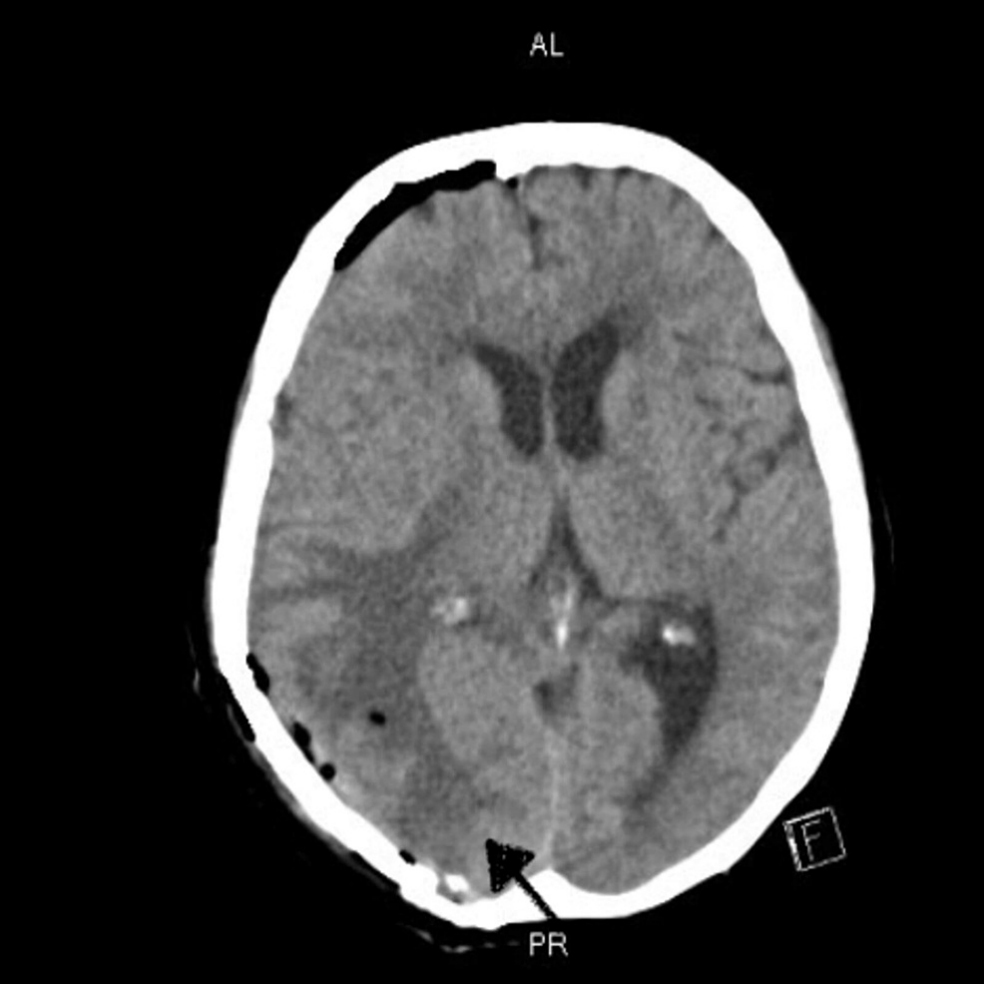
Post-Surgery CT Scan of the Head (Axial View) Without Any Evidence of Mass, Mass Effect, Midline Shift or Hemorrhage

A postoperative CT head was negative for any mass, mass effect, midline shift, hemorrhage, or large vascular territorial acute infarct (Figure 5).

She was doing well post surgery, her physical exam was at baseline. Three days after the surgery she was discharged to a rehabilitation facility with a recommendation to continue dexamethasone 4 mg every 6 hours. Three weeks after surgery, she was seen by medical and radiation oncology team for discussion regarding the initiation of systemic therapy. On oncology evaluation she complained of persistent left-sided visual field deficits, however no further episodes of persistent headaches since her surgery. She continued to have quite a significant gait ataxia and was ambulating using a walker at home, and a wheelchair while going out of the home. On examination, she was ill-appearing, lethargic, minimally verbal, and wheelchair-bound.

As primary CNS lymphoma is an extremely rare tumor with generally aggressive biology compared to nodal lymphomas there was discussion regarding all the treatment options including combination chemotherapy. Generally, the mainstay of treatment is induction therapy with intensive combination chemotherapy using high-dose methotrexate, typically in combination with anti-CD20 agent rituximab or other chemotherapy agents, followed by consolidation with either whole brain radiation or autologous stem cell transplant in select individuals. These types of treatments are typically administered in the inpatient and require quite a frequent lab monitoring of drug levels. As these treatments were not available at our institution patient was referred to the larger tertiary care center.

Other alternatives for induction, especially in elderly patients with poor performance status, include either rituximab + temozolomide or whole brain radiation therapy (WBRT) as monotherapy. We discussed that the data for rituximab + temozolomide is mostly retrospective and studied in patients with progressive, refractory disease, and there is little or no data to the upfront setting, however, it is a reasonable option to consider in patients who were otherwise ineligible for more intensive approach. Alternatively, local therapy with WBRT can be utilized, although it is associated with long-term neurotoxicity, and may be reserved for the second-line setting. After extensive discussion with the patient and family about all the treatment options, the patient declined the second opinion and agreed with starting treatment with temozolomide and rituximab, using a similar dosing schedule as was used in the relapsed/refractory setting (Temozolomide 150 mg/m^2^ once daily for five days every 28 days, initially in combination with rituximab (for four cycles), followed by temozolomide monotherapy: 150 mg/m^2^ once daily for five days every 28 days for eight cycles).

The patient was still deciding on treatment options and came for follow-up with oncology in one month. Since her last visit, she had a progressive functional decline, had three hospital admissions with failure to thrive, weakness. She was requiring significant assistance with any degree of activity. She was wheelchair bound, had been experiencing significant difficulty performing any tasks, including standing up from her wheelchair. Given the patient's relative frailty and inability to travel to a facility where she will be treated with high-dose methotrexate, a plan was made to continue with a less aggressive alternative like rituximab, temozolomide, and lenalidomide. The patient's functional status continued to decline, and any treatment moving forward was unlikely to provide any clinically meaningful benefit; hence the goal of care was discussed, focusing on prognosis, aggressive treatment vs. pursuing hospice care. After informed discussion, the family and the patient decided to continue with hospice.

## Discussion

Primary CNS lymphoma (PCNSL) is a rare disease that comprises 0.8% to 6.6% of all primary CNS tumors [3]. PCNSL had an annual incidence of 0.43 per 100,000 from 2009 to 2013 in the general population [4]. There is a male preponderance with a male to female ratio of 1.25 [5]. It is more common in Caucasians than African Americans, the ratio being 1.33 [4,5]. More than 90% of PCNSL are diffuse large B cell lymphoma (DLBCL), so PCNSL and DLBCL are sometimes used interchangeably. They exhibit Bcl6 over-expression with ongoing mutational activity [6]. The primary T-cell CNS lymphomas are approximately four percent only [7]. The WHO classification of PCNSL is depicted in Table 1.

**Table 1:** Classification of Primary Central Nervous System Lymphoma (PCNSL) AIDS = Acquired immunodeficiency syndrome; CNS = Central nervous system; NK = Natural killer

WHO Classification of PCNSL (2016)
Diffuse large B-cell lymphoma of the CNS
Immunodeficiency-associated CNS lymphomas; AIDS-related diffuse large B-cell lymphoma; Epstein-Barr virus-positive diffuse large B-cell lymphoma; Lymphomatoid granulomatosis; Post-transplant lymphoproliferative disorder
Intravascular large B-cell lymphoma
Miscellaneous rare CNS lymphomas; low-grade B-cell lymphoma; T-cell and NK-/T-cell lymphoma; Anaplastic large cell lymphoma
Malt lymphoma of the dura

Different molecular mechanisms are involved in the pathogenesis of PCNSL. Different mechanisms target different genes, which become dysregulated and lead to uncontrolled B-cell differentiation and proliferation. During B-cell differentiation, primarily somatic hypermutation, DNA double-strand breaks are required; failure to do so may lead to the formation of malignant cells. One of the crucial mechanisms is translocations affecting Ig and Ig-related genes, especially Bcl6 [8]. A substitution in the promoter of the Bcl6 gene can result in tumorigenic Bcl6 activity.

PCNSL commonly develops in immunocompromised patients. People on immunosuppressants, HIV/AIDS positive patients, and those with organ transplantation are at a higher risk [2]. In immunocompromised patients, the cause is invariably related to EBV infection, usually detected by immunohistochemistry or polymerase chain reaction. In contrast, EBV is rarely seen in PCNSL in immunocompetent patients [9]. The pathogenesis in immunocompetent patients remains uncertain.

The most common clinical feature of PCNSL includes dizziness and ataxia. Some patients may initially complain of dizziness and weakness without any focal neurological involvement, similar to our patient’s presentation [5]. Other presenting features include headache, seizure, vomiting, clumsiness, and problems with vision that progressively worsen.

The diagnosis of PCNSL relies on relevant clinical features, radiological investigations, and tissue biopsy. MRI appearance of PCNSL is characterized by homogenous hypo or isodense (T1 weighted image) and iso or slightly hyperdense (T2 weighted image) signals [10]. The PCNSL are solitary and usually >1 cm at the time of diagnosis.

The metastatic tumor of the brain usually shows significant surrounding edema relative to the size of a tumor, peripheral enhancing lesion, and approximately 30% being a solitary lesion [11]. Diagnosis of PCNSL is often delayed due to initial consideration of more common tumors like secondary CNS tumors or benign CNS tumors, quite similar to our case report [12].

The treatment of PCNSL with surgery has been debated [13]. The first primary CNS anaplastic diffuse large-B cell lymphoma patient experienced rapid deterioration after surgery and initiation of corticosteroid therapy and died three months after admission [14]. Tumor resection may lead to progression-free survival in patients with single lesions [15]. However, surgery has a poorer response if deeper brain structures are involved. The first-line treatment of PCNSL is systemic chemotherapy which involves high dose methotrexate-based chemotherapy [16]. Combination therapy with other chemotherapeutic agents that cross the blood-brain barrier, such as high-dose cytarabine, thiotepa, or ifosfamide, increases the overall response rate. However, toxicity is also increased while treatment-associated mortality remains unchanged [17]. Adding rituximab to the therapy further improves treatment response. Also, tumor control is significantly achieved when radiotherapy is combined with chemotherapy [16].

Important prognostic factors of the PCNSL are given by the International Extranodal Lymphoma Study Group (IELSG), as shown in Table 2 below [18].

**Table 2:** IELSG Prognostic Scoring for PCNSL CSF = Cerebrospinal fluid, ECOG = Eastern cooperative oncology group, LDH = Lactate dehydrogenase, PS = Performance Status, IELSG = International Extranodal Lymphoma Study Group, PCNSL = Primary central nervous system lymphoma.

Variables	Favourable Score (Value 0)	Unfavourable Score (Value 1)	Score	Two years survival
Age	= 60	>60	Low (0-1)	80%
ECOG PS	0-1	>1		
LDH	Normal	Elevated	Intermediate (2-3)	48%
CSF protein	Normal	Elevated		
Deep brain structure involvement	Involved	Not involved	High (4-5)	15%

## Conclusions

Due to consideration of more common CNS tumors, the diagnosis of PCNSL is usually delayed leading to unfavorable outcomes. When indefinite, about the diagnosis, the biopsy is recommended. Early diagnosis and management are of great importance. Although combined surgery and chemotherapy and radiotherapy are recommended, the role of surgery should be reconsidered depending upon factors such as age, performance status, and deep brain structure involvement.
